# Admission prevalence of deep vein thrombosis in elderly Chinese patients with hip fracture and a new predictor based on risk factors for thrombosis screening

**DOI:** 10.1186/s12891-018-2371-5

**Published:** 2018-12-20

**Authors:** Fei Xing, Lang Li, Ye Long, Zhou Xiang

**Affiliations:** Department of Orthopaedics, West China Hospital, Sichuan University, No. 37 Guoxue Lane, Chengdu, 610041 Sichuan People’s Republic of China

**Keywords:** Hip fracture, Deep vein thrombosis, Predictor, D-dimer, Risk factors

## Abstract

**Background:**

Elderly hip fracture (HF) patients are at very high risk of developing deep vein thrombosis (DVT), which increases their perioperative mortality. However, data focusing on the admission prevalence of DVT in elderly Chinese patients with hip fracture are limited. Venography and ultrasonography are not suitable for most elderly HF patients; there is also controversy about the prognostic value of D-dimer in elderly patients. Thus, our primary goal was to clarify the prevalence of and risk factors for DVT in elderly Chinese HF patients at admission. Our secondary goal was to evaluate the diagnostic value of a new predictor of DVT based on the risk factors for elderly HF patients.

**Methods:**

This retrospective study was conducted in the West China Hospital, Sichuan University. Between January 2015 and January 2017, 248 elderly Chinese HF patients (> 60 years) were enrolled in this study. The subjects were diagnosed with DVT using ultrasonography or venography. All the patients’ clinical data were obtained, including demographic variables, medical history, comorbidities, and laboratory results. A stepwise multiple logistic regression analysis was used to identify the risk factors contributing to the occurrence of DVT. The value of the new DVT predictor was calculated using a formula based on the coefficient regression and independent variables. A receiver operating characteristic (ROC) curve analysis was used to determine the diagnostic value of different factors.

**Results:**

Of the study patients, 74 (29.8%) were diagnosed with DVT, including sixty-five (87.8%) with distal peripheral, five (6.8%) with proximal central and four (5.4%) with mixed DVT. A multivariate logistic regression analysis showed that five risk factors increased the occurrence of DVT at admission, including gender, age, time from injury to admission, fibrinogen, and D-dimer. The new DVT predictor was calculated using the following formula: 1.131× (female = 1, male = 0) + 0.071 × age (years) + 0.571 × time from injury to admission (days) + 1.028 × fibrinogen(g/L) + 0.123 × D-dimer(g/L). The diagnostic value of the new predictor was highest among those risk predictors whose AUC (area under the ROC curves) value was 0.852.

**Conclusions:**

The results of this study revealed a high prevalence of DVT in elderly Chinese HF patients at admission. Moreover, the new predictor, based on risk factors, was a good method to improve the diagnosis of DVT.

## Background

Hip fracture, especially in elder patients, is associated with increased disability and mortality [1]. Moreover, hip fracture is a strong risk factor for DVT. DVT, an acute disease, is the formation of deep vein blood clots. The thrombus that sheds off can cause pulmonary embolism (PE), which remains the third most common cause of death in patients surviving the first 24 h of trauma [2, 3]. PE imposes a heavy medical-economic burden in many developing countries. In HF patients, DVT or PE is one of the most severe complications that can occur and increases the perioperative mortality [4]. A previous study revealed that 30% of preoperative patients with femoral neck fracture in China were diagnosed with DVT [5]. However, the incidence of perioperative DVT in Chinese patients with fracture is currently unclear [5, 6]. In addition, data on the admission prevalence of DVT in elderly Chinese HF patients are rare. In our hospital, research about the prevalence of DVT in patients with fractures have begun in recent years. Therefore, more researches are needed to figure out the prevalence of DVT in elderly Chinese HF patients. The risk factors associated with DVT are well known, including pregnancy, obesity, malignancy, respiratory failure, trauma, and nephritic syndrome [7, 8]. However, there is little current research on the risk factors for DVT in elderly Chinese HF patients.

It is widely accepted by orthopaedic surgeons that early operation for HF patients decreases the risk of perioperative complications and death [9, 10]. However, many orthopaedic surgeons have to prolong the preoperative waiting time of HF patients to determine if the patients have DVT [11]. By routinely scanning all of these HF patients, many orthopaedic surgeons could figure out if there exists DVT. In addition, some DVTs are occult and silent, which might never cause a problem. Many routinely auxiliary examinations would postpone surgery. Currently, DVT diagnosis is mostly performed using plasma markers, venography and colour Doppler ultrasonography. Although it is the gold standard to diagnose venous thromboembolism, the use of venography is limited in clinical work because of its invasiveness and non-repeatability [12]. In recent years, ultrasonography is becoming clinically more popular because it is a non-invasive and repeatable procedure [13]. However, ultrasonography requires the changing of positions, which was not suitable for fracture patients, especially those patients with hip fractures. The accuracy of ultrasonography is also influenced by the expertise of the performing physician. In addition, facilities for venography or ultrasonography may not be widely available in primary hospitals in developing Asian countries. Plasma markers are commonly used to diagnose DVTs. As the final product of the plasmin-mediated degradation of cross-linked fibrin, D-dimer is a useful diagnostic test for patients with suspected DVT [14, 15]. However, there is controversy about the diagnostic value of D-dimer in elderly patients [16, 17].

The primary goal of the present study was to clarify the admission prevalence and risk factors for DVT in elderly Chinese patients with hip fracture. The secondary goal of the study was to assess the effectiveness of a new DVT screening method for elderly HF patients.

## Methods

**Fig. 1:**
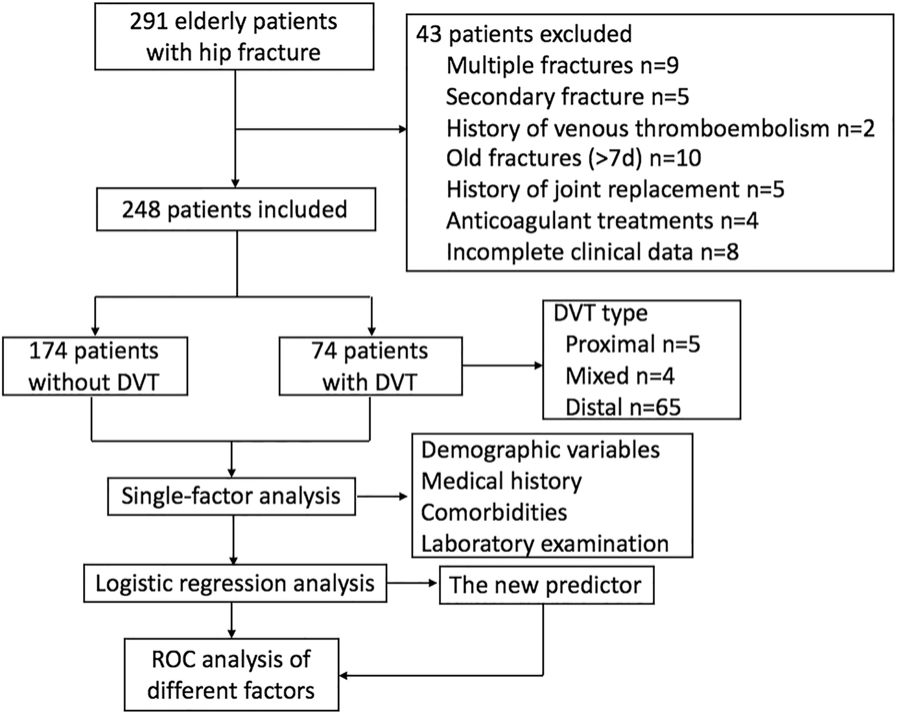
The sampling procedure used for all elderly patients with hip fracture in this study

This retrospective study was approved by the institutional ethical review board of our institution (West China Hospital, Sichuan University, Chengdu, China). Two hundred and ninety-one patients older than 60 years were diagnosed with hip fracture at West China Hospital between January 2015 and January 2017. The exclusion criteria used were: secondary fracture, multiple fractures, previous history of venous thromboembolism, old fracture (>7d), history of joint replacement, anticoagulant treatments, incomplete imaging or laboratory test results and other miscellaneous reasons. Overall, forty-three patients were excluded from the study. The case data collection was completed by several clinicians who had received a standardized training. A total of two hundred and forty-eight elderly HF patients were included in this study. Among these patients, two hundred and thirty-five patients had undergone surgeries by professionally trained orthopaedists. Of these, 131 patients were treated with proximal femoral nail anti-rotation (PFNA), 59 patients were treated with joint arthroplasty, 29 patients were treated with a dynamic hip screw (DHS), and 16 patients were treated with a hollow compression screw. The patients were diagnosed with DVT using venography or colour Doppler ultrasonography. Colour Doppler ultrasonography was conducted by experienced radiologists in a colour ultrasonic room. All results of the ultrasonography were reviewed by senior radiologists. Re-examination of ultrasonography occurred when there were different opinions. All elderly HF patients without DVT received DVT prophylaxis, including physical or pharmacological thromboprophylaxis (a hypodermic injection of low molecular heparin). In the absence of clinical symptoms of DVT, including unilateral lower limb sudden swelling and pain, venography or ultrasonography were performed again. Patients diagnosed with DVT received anticoagulation and thrombolytic therapy. All the HF patients underwent venography or ultrasonography at the same time as their laboratory tests. The clinical patient data obtained included demographic variables (gender, age, body height, body weight, BMI, injury side, injury mechanism, and time from injury to admission), medical history (smoking, cerebrovascular disease, and malignancy), comorbidities (including hypertension, chronic obstructive pulmonary disease, diabetes, arrhythmia, renal dysfunction, Parkinson’s disease, varicose veins, and coronary heart disease), laboratory tests (haemoglobin, blood platelet, serum albumin, serum sodium, serum potassium, fibrinogen, prothrombin time, thrombin time, activated partial thromboplastin time, and D-dimer). The sampling procedure of all the elderly patients with hip fracture in this study is shown in Fig. 1.

In single-factor analysis, the patients were divided into two groups: with and without DVT. The two groups were compared to determine which independent variables showed significant differences between the two groups. A stepwise multiple logistic regression analysis was used to identify which independent variables contributed to the occurrence of DVT. Statistical variables were input in a logistic regression analysis to determine the coefficient regression (CR) of the independent variables. The DVT predictor value was calculated using a formula based on the coefficient regression and the independent variables. A receiver operating characteristic curve (ROC) analysis was used to determine the diagnostic value of statistically significant factors based on the sensitivity and specificity of the DVT detection results.

We used the software Statistical Product and Service Solutions (IBM SPSS Inc., Version 21, Chicago, USA) for statistical analysis, and a statistically significant difference was assumed if *p*-values were less than 0.05. Continuous variable data were reported as the mean value and the standard deviation and compared using either an independent t-test or a Mann-Whitney U test, as appropriate. Categorical variables were summarized using proportions and compared using the chi-square test. A logistic regression analysis was used to generate adjusted odds ratios and to investigate whether factors with significant differences contributed to the occurrence of deep vein thrombosis. Factors with significant differences were further analysed using multiple logistic regression to generate adjusted odds ratios. ROC curves analyses were also used to analyse the results, and the sensitivity and specificity were calculated as different cut-off values of statistically significant factors.

## Results

A total of 248 elderly Chinese patients (94 males and 154 females) admitted to hospital with hip fracture were enrolled in this retrospective study. The demographics of the patients are shown in Table 1. Of the study patients, 74 (29.8%) were diagnosed with DVT by venography or ultrasonography. Of these 74 patients, sixty-five (87.8%) had distal peripheral DVT, five (6.8%) had proximal central DVT and four (5.4%) had mixed DVT. Two of the patients with distal DVT subsequently developed mixed DVT. None of the patients developed pulmonary artery embolism. Of the patients without DVT at admission, five were diagnosed with DVT whilst awaiting surgery. Further comparison of the patients with or without DVT showed statistically significant gender differences. The mean age of the enrolled patients was 78.72 ± 8.68 years (ranging from 60 to 98 years). The mean time from injury to admission was 1.91 ± 1.52 days. The age and time from injury to admission were both significantly higher in the DVT group than in the group without DVT. Of the 248 HF patients, 121 had intracapsular fractures; the rest had extracapsular fractures. A total of 116 patients had intertrochanteric fractures and 11 had subtrochanteric fractures. There was no difference between the two groups. Ten patients had high energy hip fractures; the rest of the patients had low energy fractures. Fourteen patients had a history of cerebrovascular disease. Six patients had a history of malignancy. Comorbidities in the HF patients included 104 patients with hypertension, 69 patients with chronic obstructive pulmonary disease, 53 patients with diabetes, 31 patients with arrhythmia, 28 patients with coronary heart disease, 7 patients with Parkinson’s disease, 7 patients with renal dysfunction, and 4 patients with varicose veins. Except for Parkinson’s disease, the comorbidity rate was higher in the DVT group than in the group without DVT. However, the only significant difference in the comorbidity rate between the two groups was in the rate of diabetes. The mean amount of haemoglobin in the HF patients was 109.22 ± 11.7 g/L. The average levels of serum albumin, serum potassium, and serum sodium in the HF patients were 32.04 ± 3.43 g/L, 4.11 ± 0.51 mmol/L, and 138.56 ± 3.88 mmol/L, respectively. The blood coagulation times of the HF patients are shown in Table 1. The mean levels of D-dimer and fibrinogen were 7.57 ± 6.44 g/L and 4.33 ± 0.74 g/L, respectively. The DVT group had significantly higher levels of plasma D-dimer and fibrinogen than the group without DVT.

**Table 1:** Patient demographics associated with DVT on admission

Variable	Total Patients (*n* = 248)	Patients with DVT (*n* = 74)	Patients without DVT (*n* = 174)	*P* value*
Demographic Variables				
Gender (Male/female)	94/154	20/54	74/100	0.021
Age (years)	78.72 ± 8.68	81.74 ± 7.70	77.43 ± 8.77	<0.001
Body height (m)	1.59 ± 0.07	1.58 ± 0.07	1.60 ± 0.07	0.116
Body weight (kg)	54.89 ± 9.87	54.83 ± 9.56	54.91 ± 10.03	0.956
BMI (kg/m^2^)	21.54 ± 2.98	21.81 ± 2.71	21.42 ± 3.09	0.346
Time from injury to admission (days)	1.91 ± 1.52	2.50 ± 1.82	1.66 ± 1.30	<0.001
Injury side (left/right)	146/102	44/30	102/72	0.902
High energy mechanism	10	4	6	0.716
Medical history				
Smoking history (current or past)	100	34	66	0.239
History of cerebrovascular disease	14	4	10	0.915
History of malignancy	6	3	3	0.522
Comorbidities				
Hypertension	104	37	67	0.093
Chronic obstructive pulmonary disease	69	26	43	0.094
Diabetes	53	23	30	0.015
Arrhythmia	31	10	21	0.753
Coronary heart disease	28	10	18	0.471
Parkinson’s disease	7	2	5	0.941
Renal dysfunction	7	3	4	0.460
Varicose veins	4	2	2	0.395
Laboratory Examinations				
Hemoglobin(g/L)	109.22 ± 11.7	107.25 ± 10.29	110.06 ± 12.18	0.064
Blood platelet(10^9/L)	173.78 ± 56.78	175.08 ± 55.15	173.23 ± 57.60	0.815
Serum albumin(g/L)	32.04 ± 3.43	31.44 ± 3.57	32.30 ± 3.34	0.068
Serum potassium(mmol/L)	4.11 ± 0.51	4.16 ± 0.46	4.09 ± 0.53	0.321
Serum sodium(mmol/L)	138.56 ± 3.88	138.77 ± 3.91	138.47 ± 3.88	0.576
Fibrinogen(g/L)	4.33 ± 0.74	4.52 ± 0.71	4.24 ± 0.74	0.007
PT(s)	11.99 ± 0.95	12.12 ± 1.05	11.94 ± 0.90	0.175
TT(s)	17.18 ± 1.09	17.00 ± 1.13	17.24 ± 1.06	0.106
APTT(s)	29.78 ± 2.24	29.54 ± 2.17	29.88 ± 2.26	0.269
D-dimer (μg/ml)	7.57 ± 6.44	9.84 ± 6.84	6.59 ± 6.02	<0.001

*The chi-square test or Independent t test was conducted to compare inter-group difference

**Fig. 2:**
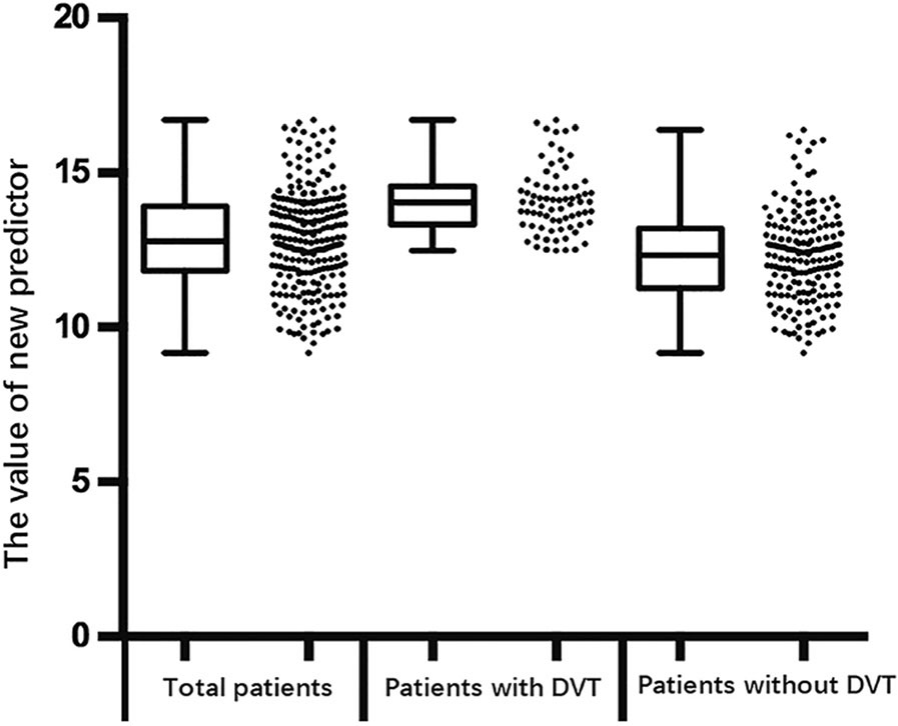
A scatter dot plot of the new DVT predictor values of the DVT and non DVT groups

Single-factor analysis indicated that gender, age, time from injury to admission, D-dimer, fibrinogen, and diabetes were significantly associated with the occurrence of DVT in elderly patients admitted to hospital with hip fracture. The results of the univariate logistic regression analysis showed that only five factors (gender, age, time from injury to admission, D-dimer, and fibrinogen) were significantly (*P* < 0.05) associated with DVT in hospital-admitted elderly HF patients. Multivariate logistic regression analysis confirmed the five significant risk factors for DVT in hospital-admitted elderly HF patients as gender (OR 2.895, *P* = 0.005), age (OR 1.074, *P* = 0.001), time from injury to admission (OR 1.752, *P* < 0.001), fibrinogen (OR 2.747, P < 0.001), and D-dimer (OR 1.131, P < 0.001). There was no intergroup statistical significance in the odds ratio of diabetes. **(**Table 2**)** Therefore, in elderly Chinese HF patients, gender (CR = 1.133), age (CR =0.071), time from injury to admission (CR = 0.571), fibrinogen (CR = 1.028), and D-dimer (CR = 0.123) could be used to predict the occurrence of DVT at admission. **(**Table 3**)** A new DVT predictor was calculated using the following formula: The value of new predictor = 1.131× (female = 1, male = 0) + 0.071 × age (years) + 0.571 × time from injury to admission (days) + 1.028 × fibrinogen (g/L) + 0.123 × D-dimer (g/L). The mean new predictor value of the enrolled patients was 12.84 ± 1.59. The mean new predictor value of the DVT group (14.11 ± 1.08) was significantly higher (*P* < 0.001) than the predictor value of the group without DVT (12.30 ± 1.47). A scatter dot plot of the new DVT predictor values of the DVT and non DVT groups is shown in Fig. 2.

**Table 2:** The results of univariate logistic regression analysis for DVT

Risk Factors	OR	95% CI	*P*-value
Gender	2.895	1.374–6.100	0.005
Age (years)	1.074	1.029–1.121	0.001
Time from injury to admission (days)	1.752	1.393–2.204	< 0.001
Diabetes	1.947	0.897–4.229	0.092
Fibrinogen(g/L)	2.747	1.636–4.613	< 0.001
D-dimer (μg/ml)	1.131	1.073–1.193	< 0.001

**Table 3:** The results of multiple logistic regression analysis for factors with significant difference

Risk Factors	Coefficient regression	SE	Wald	OR	95% CI	*P*-value
Gender	1.133	0.376	9.054	3.104	1.484–6.493	0.003
Age (years)	0.071	0.022	10.419	1.073	1.028–1.120	0.001
Time from injury to admission (days)	0.571	0.116	24.116	1.770	1.409–2.223	< 0.001
Fibrinogen(g/L)	1.028	0.261	15.487	2.795	1.675–4.664	< 0.001
D-dimer (μg/ml)	0.123	0.027	20.710	1.131	1.072–1.192	< 0.001

**Fig. 3:**
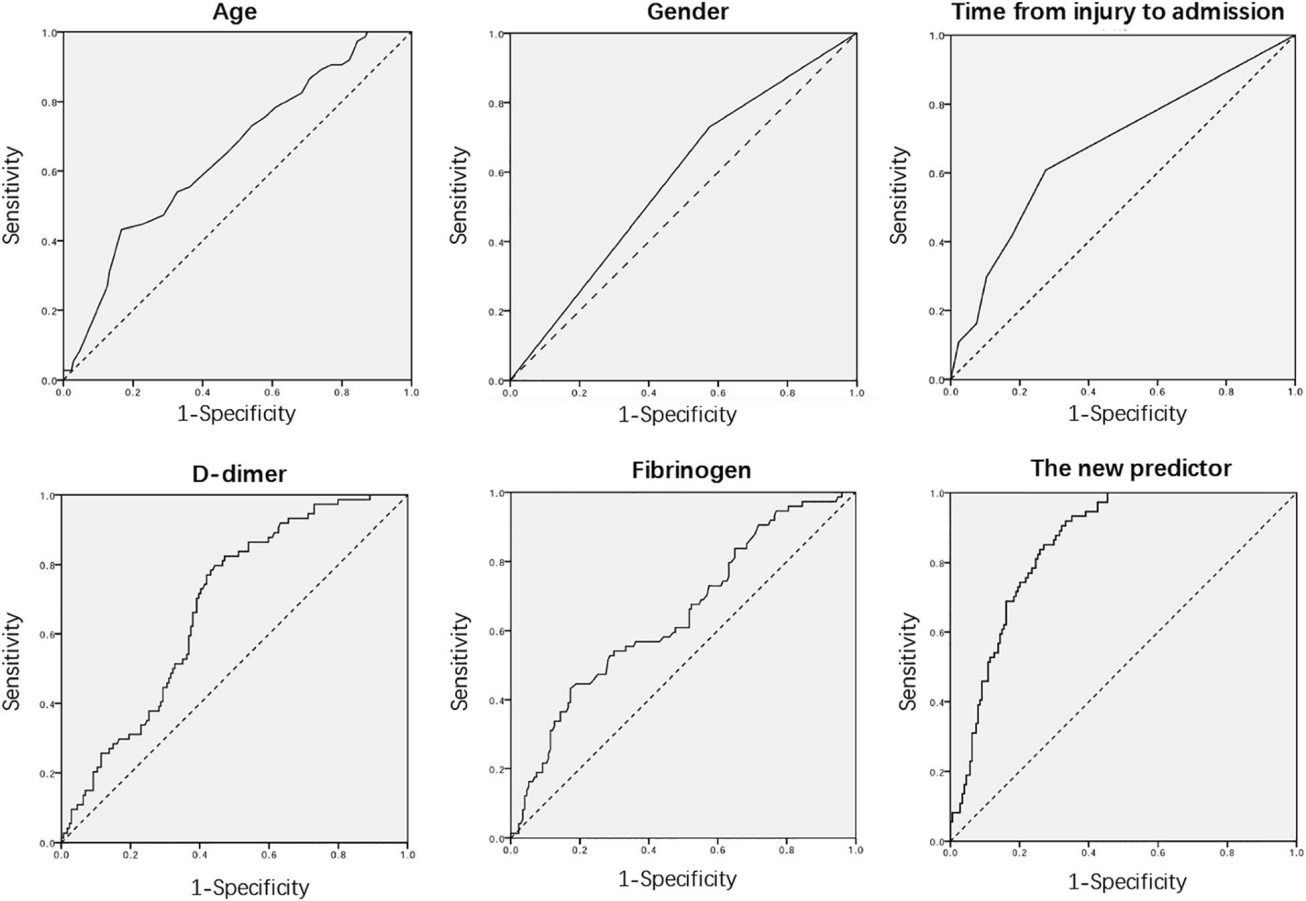
The ROC curves of different factors

The diagnostic value of the statistically significant factors was analysed using the ROC. The results of the ROC indicated that age, time from injury to admission, fibrinogen, D-dimer, and the new DVT predictor value could be used in DVT diagnosis. The ROC curves of the different factors are shown in Fig. 3**.** The value of the area under the ROC curves (AUC) indicates the diagnostic value of the predictors. The new DVT predictor value was highest among the factors whose AUC value was 0.852. The AUC value of D-dimer, age, time from injury to admission, and fibrinogen were 0.679, 0.649, 0.674, and 0.644, respectively. The diagnostic cut-off values of these predictor were calculated using Youden’s index as 12.7432 (new predictor), 5.035 (D-dimer), 84.5 (age), 1.5(time from injury to admission), and 4.89 (fibrinogen). **(**Table 4**)** Sensitivity and specificity analyses were performed for all the risk predictors at their cut-off values.

**Table 4:** The ROC results of different factors

Risk Factors	Cut-off value^a^	Sensitivity	Specificity	NPV	PPV	AUC	95% CI	SE	*P*-value
Gender	–	0.730	0.425	0.787	0.351	0.578	0.501–0.654	0.039	0.054
Age (years)	84.5	0.446	0.776	0.767	0.458	0.649	0.575–0.723	0.038	< 0.001
Time from injury to admission (days)	1.5	0.608	0.724	0.813	0.484	0.674	0.598–0.750	0.039	< 0.001
Fibrinogen(g/L)	4.89	0.432	0.828	0.744	0.516	0.644	0.569–0.719	0.038	< 0.001
D-dimer (μg/ml)	5.04	0.797	0.557	0.837	0.378	0.679	0.612–0.746	0.034	< 0.001
New predictor	12.74	0.919	0.667	0.951	0.540	0.852	0.806–0.898	0.023	< 0.001

^a^The cut-off points of scores were determined by the Youden index

## Discussion

As a heightened risk of DVT begins immediately after hip fracture, blood clot formation can happen at any time, especially in elderly patients. Previous studies showed that the incidence of preoperative DVT in HF patients was between 6 and 62%, but no study assessed the admission rate of DVT in elderly Chinese HF patients [5, 18, 19]. Previous studies also ignored the fact that many elderly HF patients had already had venous thrombosis at admission. In our study, we revealed a high incidence of DVT in elderly Chinese HF patients at admission, which indicates that many elderly Chinese HF patients on admission already have DVT. Furthermore, the DVT could be divided into occult DVT and symptomatic DVT. The high prevalence of DVT on admission might be related to occult DVT which may have never caused a problem. However, few studies have focused on the long-term effects of occult DVT in elderly Chinese HF patients. In addition, unearthing occult DVT and early intervention might reduce the incidence of symptomatic DVT. In our study, both occult or symptomatic DVT were regarded as one type of DVT. In addition, previous history of venous thromboembolism and anticoagulant treatment were both exclusion criteria in our study included, so the time of thrombosis formation was from injury to admission and the DVT patients found in our study all had acute DVT. Currently, most doctors would treat elderly HF patients with some drugs or physical methods to prevent the occurrence of DVT. However, if elder HF patients was diagnosed with DVT at admission, doctors would have to start these patient with DVT treatment immediately rather than giving DVT prophylaxis. Thus, in elderly HF patients, early DVT identification is essential to limit late complications of DVT and prevent clot extension, acute PE, and recurrent thrombosis [20].

In our study, most of patients were diagnosed with distal DVT, which was consistent with a previous study [5]. However, the clinical relevance of distal DVT in elderly HF patients is disputed. We believe that patients with distal DVT also need active treatment, as there is no long-term follow-up study on the effect of distal DVT on the rehabilitation of HF patients, especially elderly Chinese patients.

In our study, age was an independent risk factor for DVT in elderly HF patients. This may have be caused either by an acquired prothrombotic state, the anatomical variations of lower veins and the thickening of venous valve cusps [21]. Some studies demonstrated that women have a higher risk of venous thrombosis than men, while others hold opposite view [21–23]. Our study identified being female as a risk factor for DVT in elderly HF patients, which is supported by a previous study [24]. Another study found that obesity was associated with DVT [25], however, our study, found no association between BMI and DVT in elderly HF patients. D-dimer and fibrinogen are indicators of coagulation function. In our study, high level of D-dimer and fibrinogen were associated with DVT in elderly HF patients. D-dimer is used to diagnose venous thromboembolism, including deep vein thrombosis and pulmonary embolism. However, there is controversy in determining the cut-off value of D-dimer because of its low diagnostic accuracy, especially in elderly patients [11, 26]. Prolonged immobilization and delays in fracture fixation were independent risk factors for DVT noted in previous studies [5, 24, 27]. The preoperative period of immobilization could be divided into the time from injury to admission and the period awaiting surgery [19, 28]. In our study, all elderly HF patients underwent venography or ultrasonography on admission. Five patients were diagnosed with DVT in the period awaiting surgery. This suggests that only a small proportion of DVT happens between admission and surgery. In our opinion, pharmacological or physical thromboprophylaxis greatly reduced the occurrence of DVT in the period awaiting surgery. In addition, our study showed that the time from injury to admission was associated with increased risk of DVT. Thus, clinicians should pay attention to not only the time awaiting surgery but also the time from injury to admission. In our view, improving the awareness of elderly HF patients to the need for hospitalization and shortening the time to hospitalization -transferring would shorten the time from injury to admission and decrease the risk of DVT. Taking all these risk factors into account, clinicians should know an individual’s risk of DVT and make a personalized treatment programme to prevent the incidence of pulmonary embolism. In our study, DVT was diagnosed by venography or colour Doppler ultrasonography. The diagnostic accuracy of colour Doppler ultrasonography might be lower than venography. However, in our study, an experienced radiologist could effectively improve the diagnostic accuracy of colour Doppler ultrasonography. Furthermore, superior examination could also decrease the misdiagnosis rate.

Currently, DVT diagnosis is mostly performed using venography, colour Doppler ultrasonography, and plasma markers. Venography was a gold standard to diagnose DVT, but was limited by its invasiveness and non-repeatability in clinical work [12]. Ultrasonography requires specialized equipment and experienced radiologists on 24-h duty, which is not available in some primary hospitals. The results of the ultrasonography can also be affected by the proficiency of the radiologist. Moreover, elderly HF patients would suffer from the pain caused by changing the positions of lower extremities. In addition, the waiting time for venography or ultrasonography would result in delays to surgery and increase the risk of perioperative complication [18, 29]. Some patients at low risk for DVT could be treated with early operation as soon as possible instead of waiting for venography or ultrasonography. A previous study examined the usefulness of erythema, lower limb swelling, tenderness, Homan’s sign, and calf diameter in predicting DVT, but did not find that any of these factors performed well in diagnosing DVT [30]. D-dimer is used as a diagnostic tool for DVT, however, the levels of D-dimer are influenced by many factors, such as inflammation, age, surgery, hospitalization, COPD, and other acute diseases [31–33]. In addition, there is controversy about the cut-off value of D-dimer for the diagnosis of DVT in elderly patients, especially in elderly trauma patients [15, 34]. Many recent studies have attempted to use age-adjusted cut-offs for D-dimer in the diagnosis of DVT in elderly outpatients to lower the incidence of pulmonary embolism [35, 36]. However, few studies focused on adjusted cut-off values for D-dimer in elderly trauma patients, especially in elderly HF patients. Receiver operating characteristic curves (ROC) are usually used to evaluate the diagnostic value of different methods: the larger the value of the area under curve (AUC), the better the diagnostic value. In our study, we calculated the AUC of all the independent risk factors, and found that D-dimer had the highest diagnostic value. However, the AUC of D-dimer was only 0.679. Thus, our study attempted to improve the DVT diagnostic system by using a new method, adjusted by independent risk factors including gender, age, time from injury to admission, D-dimer, and fibrinogen.

Previous studies mainly reported adjusted D-dimer cut-off values by age in elderly individuals. Han et.al reported age-adjusted D-dimer cut-off value by age × 0.01 μg/ml in elderly patients (> 50 years) [15]. In another study, they also found age-dependent reference intervals for D-dimer in elderly general population [26]. In the study of Schouten et al., they calculated adjusted D-dimer in elderly outpatients by the following formula:(0.1 × age (years) × D-dimer value (g/mL) [35]. Imai et al. calculated the D-dimer index of patients before total hip arthroplasty by 0.12 × age (years) + 0.45 × the D-dimer value (g/mL) [11]. Furthermore, the previous study also found that there was a markedly substantially increase of D-dimer and fibrinogen levels in fracture patients, especially in femoral fracture patients [37]. Using the results of multivariate logistic regression analysis, our study calculated the new DVT predictor from the following formula: the value of new predictor = 1.131× (female = 1, male = 0) + 0.071 × age (years) + 0.571 × time from injury to admission (days) + 1.028 × fibrinogen (g/L) + 0.123 × D-dimer (g/L). In our study, we reported a new calculated method to predict DVT (AUC: 0.852; 95% confidence interval: 0.806~0.898; *p* < 0.001). Figure 2 is a scatter dot plot of the new DVT predictor values of the DVT and non DVT groups. When we defined the cut-off value of the new predictor as 12.74 using Youden’s index, 116 of 248 patients had values under 12.74 with a sensitivity of 91.9% and a specificity of 66.7%. When the cut-off was 12.47, 95 of 248 patients had values under 12.47 with a sensitivity of 100%, a specificity of 54.6%, a negative predictive value (NPV) of 100% and a positive predictive value (PPV) of 48.4%. When its cut-off was 12.47, the new DVT predictor could be used as a diagnosis of exclusion, i.e., when the new DVT predictor value < 12.47, there was low risk of DVT, and surgery could be performed on elderly HF patients as soon as possible without the need for any further auxiliary examination. Our new method could improve the diagnostic value of D-dimer. The new DVT predictor had highest sensitivity among these predictor factors. For clinicians, with high sensitivity and high negative predictive value, new predictor could be conducted to exclude DVT diagnosis when the value of new DVT predictor was less than the cut-off value. New predictor estimated on admission could effectively identity the patients without DVT and ensure early operation without ultrasonography or venography. Furthermore, the new DVT predictor could be used to identify elderly Chinese HF patients with high-risk of DVT and could benefit from was helpful to take treatment of early anti-coagulation treatment and a shortened preoperative time. Follow-up studies by our group will attempt to use the new predictor in the diagnosis of DVT to shorten time from admission to surgery.

The present study had several limitations. First, our study was a retrospective analysis. Second, the sample size of enrolled patients was small, and all the patients came from same hospital. In addition, for some variables, small sample size may be underpowered to show significant differences. A multi-centre large sample study would be required to validate our findings. Third, most of the DVT patients were diagnosed using ultrasonography, which may have a lower accuracy than venography.

## Conclusions

In conclusion, our findings revealed a high prevalence of DVT on admission based on the ultrasonography or venography results of elderly Chinese patients with hip fracture. Our study has also demonstrated that the independent risk factors of gender, age, time from injury to admission, D-dimer, and fibrinogen all increase the risk of DVT in elderly HF patients. Using multivariate logistic regression analysis, our study defined a new predictor based on risk factors to diagnose DVT. This new predictor greatly improved the DVT diagnostic system. With high sensitivity and low specificity, the new predictor could be used to screen for DVT in elderly HF patients on admission.

## Abbreviations

APTT: Activated partial thromboplastin time
AUC: Area under the curve
BMI: Body mass index
CI: Confidence interval
CR: Coefficient regression
DVT: Deep vein thrombosis
HF: Hip fracture
OR: Odds ratio
PE: Pulmonary embolism
PT: Prothrombin time
ROC: Receiver operating characteristic
SE: Standard error
SPSS: Statistical product and service solutions
TT: Thrombin time
